# A missense mutation in *CRYBA4* associated with congenital cataract and microcornea

**Published:** 2010-06-05

**Authors:** Guangkai Zhou, Nan Zhou, Shanshan Hu, Liming Zhao, Chunmei Zhang, Yanhua Qi

**Affiliations:** Department of Ophthalmology, Harbin Medical University the 2nd Affiliated Hospital, Harbin, Heilongjiang, China

## Abstract

**Purpose:**

To identify mutations in a Chinese family with congenital cataract and microcornea.

**Methods:**

Detailed family history and clinical data were recorded. Genomic DNA was extracted from leukocytes of venous blood of the patients and noncarriers in this family along with 100 normal individuals. All six exons of crystallin, beta A4 gene (*CRYBA4*) were amplified by PCR methods and direct sequencing.

**Results:**

We identified a c.225G>T sequence change that led to an amino acid substitution G64W in the *CRYBA4*-induced protein in two patients of this family; this nucleotide substitution was not detected in the other individuals.

**Conclusions:**

A novel missense mutation in *CRYBA4* was identified in our study. It expands the mutation spectrum of *CRYBA4* and provides useful information to the study of molecular pathogenesis of cataract and microcornea.

## Introduction

Congenital cataract can be defined as lens opacification presenting at birth or developing shortly thereafter. The lens alone may be involved, and this accounts for approximately 70% of congenital cataracts. Conversely, lens opacities may be associated with other ocular anomalies, such as microphthalmia, aniridia, other anterior chamber developmental anomalies, or retinal degenerations, seen in approximately 15% of case [1]. Congenital cataract is a leading cause of childhood blindness worldwide and results in about 10%–20% of children in developing countries to be blind [2]. Worldwide, 20 million children under the age of 16 suffer from cataract, and among these, 200,000 (10%) are severely visually impaired or blind. While this figure is relatively low compared to the 17 million (40%) adults who are blind caused by cataract [3-5].

Recently, more than 34 loci in the human genome have been reported to be associated with congenital cataract, and 22 specific genes have detected mutations, including encoding crystallins (crystallin, alpha A gene [*CRYAA*], crystallin, alpha B gene [*CRYAB*], crystallin, beta A1 gene [*CRYBA1*], crystallin, beta A4 gene [*CRYBA4*], crystallin, beta B1 gene [*CRYBB1*], crystallin, beta B2 gene [*CRYBB2*], crystallin, beta B3 gene [*CRYBB3*], crystallin, gamma C gene [*CRYGC*], crystallin, gamma D gene [*CRYGD*], and crystallin, gamma S gene [*CRYGS*] [6-14]), cytoskeletal proteins (beaded filament structural protein 1, filensin gene [*BFSP1*], and beaded filament structural protein 2, phakinin gene [*BFSP2*] [15,16]), membrane proteins gap junction protein, alpha 3 gene (*GJA3*) and gap junction protein, alpha 8 gene (*GJA8*), major intrinsic protein of lens fiber gene (*MIP*) and lens intrinsic membrane protein 2 gene (*LIM2*) [17-20]), transcription factors (heat shock transcription factor 4 gene [*HSF4*], paired-like homeodomain 3 gene [*PITX3*], and Maf-like protein gene [*MAF*] [21-23]), glucosaminyl (N-acetyl) transferase 2 gene (*GCNT2*) [24], chromatin modifying protein-4B gene (*CHMP4B*) [25], and transmembrane protein 114 gene (*TMEM114*) [26].

We report a novel missense mutation in *CRYBA4* after analyzing a Chinese family with congenital cataract and microcornea. This mutation was not observed in any of the healthy family members.

## Methods

### Clinical evaluations

**Figure 1:**
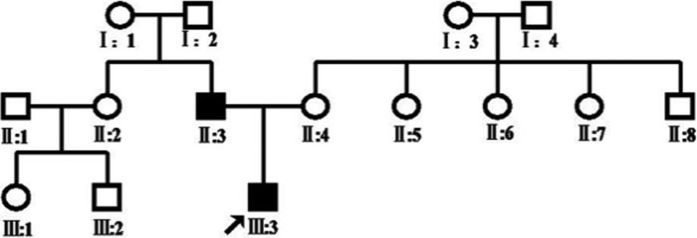
Pedigree of a Chinese family with congenital cataract and microcornea. The proband (III:3) is indicated by an arrow. Two members (II:3 and III:3) in two generations were affected with congenital cataract and microcornea. Both of the two affected members had operations for bilateral congenital cataract.

**Figure 2:**
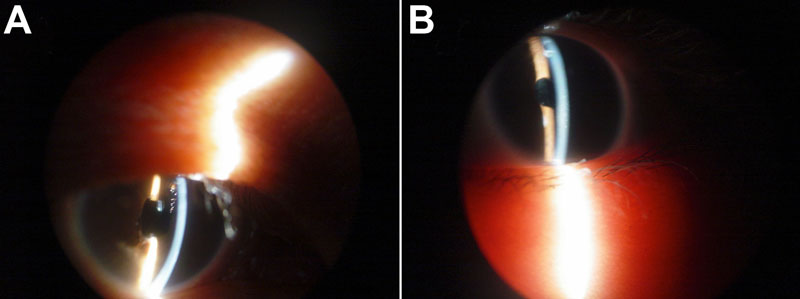
Post-operation eye photographs of the two patients with congenital cataract and microcornea in a Chinese family. There were two patients in this family we studied with congenital cataract and microcornea. They had a cataract extraction in another hospital, which provided us with post-operation photos. **A**: Post-operation eye photographs of the proband’s father is shown. **B**: Post-operation eye photographs of the proband is displayed.

A three-generation Chinese pedigree that consists of 15 individuals, including two affected individuals, provided the basis for the study. Nine family members participated in the study (two affected and seven unaffected individuals; Figure 1). Two patients (both male) in this pedigree had congenital cataract and microcornea , and had shown symptoms of vision decrease before two years old. The proband was a 7-year-old boy who had a cataract extraction in another hospital, which provided us with post-operation photos (Figure 2). According to his medical records, this patient has congenital nuclear cataract with microcornea. The axial length of his eyes is 23.4 mm oculus dexter (OD) and 24.2 mm oculus sinister (OS); the corneal diameter is 9.5 mm. His father also has congenital nuclear cataract (post operation), and the axial length of his eyes is 24.6 mm (OD) and 25.2 mm (OS); the corneal diameter is also 9.5 mm. The corneal diameter and eye axial length of seven healthy members of this family were normal (Table 1). None of the family members had any other ocular or systemic abnormalities identified after a complete physical and ophthalmologic examination. One hundred normal controls (54 males, 46 females, age 2–42 years) were recruited from Physical Examination Center of Harbin Medical University the 2nd Affiliated Hospital, Harbin, Heilongjiang, China.

**Table 1:** Corneal diameter and eye axial length of nine members of the family.

**Member**	**Corneal diameter**	**Eye axial length (OD)**	**Eye axial length (OS)**
III:3	9.5 mm	23.4 mm	24.2 mm
II:3	9.5 mm	24.6 mm	25.2 mm
I:1	11.6 mm	24.3 mm	24.7 mm
I:2	11.6 mm	23.9 mm	24.1 mm
II:2	11.8 mm	24.2 mm	25.1 mm
II:4	11.6 mm	24.7 mm	24.6 mm
II:5	11.5 mm	24.5 mm	24.1 mm
III:1	11.6 mm	24.4 mm	24.5 mm
III:2	11.5 mm	24.2 mm	24.7 mm

Two members of this family (II:3 and III:3) in two generations were affected with congenital cataract and microcornea. Other seven members(I:1, I:2, II:2, II:4, II:5, III:1, and III:2) were not involved.

The family members were interviewed to obtain a detailed medical, ophthalmic, and family history after obtaining informed consent. This study was approved by the Institutional Review Board of Harbin Medical University, Harbin, China.

### Molecular genetic studies

Peripheral blood samples (5 ml) were taken from nine members (two affected and seven unaffected individuals) of the family and 100 healthy controls, and were preserved at -20 °C in EDTA, and we then used the TIANamp Blood DNA kit (Tiangen Biltech Co. Ltd., Beijing, China) to extract genomic DNA. All six exons of *CRYBA4* were amplified by PCR using the primers listed in Table 2. The PCR products were purified and sequenced by Shanghai Invitrogen Biotechnology Co. LTD (Shanghai, China). The data were compared with sequences from the NCBI GenBank (*CRYBA4*: NM_001886), and the modeled structures were built using Swiss-PdbViewer 4.0.1 (Torsten Schwede et al.,Basel, Switzerland) [27].

**Table 2:** Primers for mutational screening of *CRYBA4*.

**Exon**	**Forward primer**	**Reverse primer**	**Product length (bp)**
1	GTCCTTTCCCTCCCTGCTAA	AGGATGAGGATGGCATTCAG	316
2	TAGCCCAGTCACTCCTGGAC	CCTAGGATTCATGGGGACCT	238
3	TTTGCAATCCCTGCTTTACC	CTTCAGGAGGGCACAACAGT	350
4	ACCCCTGAATGGTTGTGACT	CTTGAAGTGGCGACATGAGA	350
5	CAAATGGCAAGGTTTCTGGT	GTCCCTCAAATTCTGCCTGA	465
6	AGGGAATGGCATGATCAAAG	GGCCTGAAGTAAATAGAAGAAAGG	633

Summary of the primers used for the amplification of *CRYBA4* exons. Sequences are given in the 5′→3′direction.The primers were designed on line using primer 3.0.

## Results

**Figure 3:**
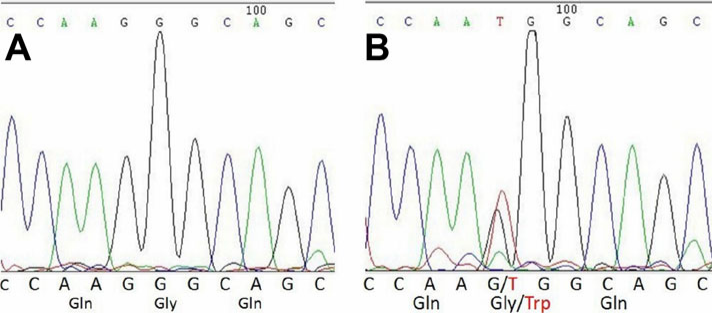
Sequence analysis of the unaffected members and affected members in the family with congenital cataract and microcornea. **A**: The partial sequence of *CRYBA4* in a normal individual is displayed. **B**: The corresponding nucleotide sequence of *CRYBA4* in the proband is shown. Sequencing results showed a substitution of G→T in *CRYBA4* (c.225G>T), which led to replacement of glycine by tryptophan at codon 64(G64W) in crystallin, beta A4 protein.

**Figure 4:**
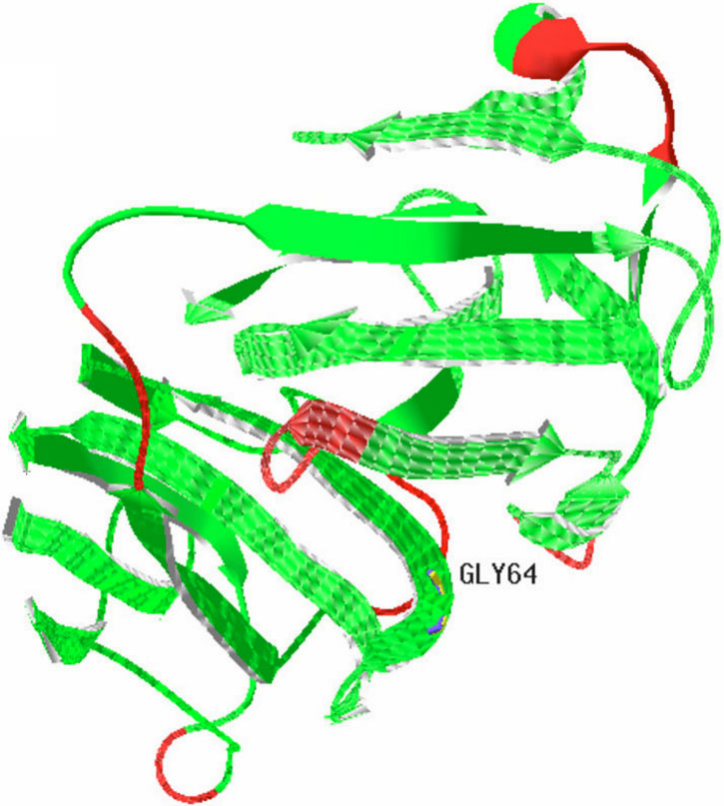
The modeled structure of crystallin, beta A4 protein (CRYBA4). The modeled structure of CRYBA4  was built using special software (Swiss-PdbViewer 4.0.1 [39-41]). The mutation described in this study leads to the replacement of glycine by tryptophan at codon 64 (Gly64).The Gly64, located in the corner of the modeled structure, may form such secondary structures as β-sheet or β-turn.

**Figure 5:**
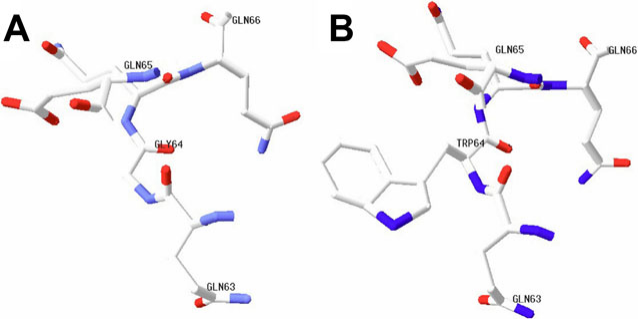
Portion of the crystallin, beta A4 protein (CRYBA4) modeled structure in the vicinity of residue 64. The portion modeled structures of the CRYBA4 in the vicinity of residue 64 were built using Swiss-PdbViewer 4.0.1 [39-41]). **A**: The normal modeled structure of CRYBA4 is displayed. **B**: The mutant modeled structure of CRYBA4 is shown. Glycine (Gly) is replaced by tryptophan (Trp) at codon 64. Modeled structures are shown by element type, using a default standard CPK (a popular color convention for distinguishing atoms of different chemical elements) scheme: n=blue, O=red, C=white.

In this family, two patients showed the same clinical symptoms, congenital nuclear cataract and microcornea, and we identified a new mutation (c.225G>T, Figure 3) in exon 4 after direct sequencing of *CRYBA4*. This mutation was not detected in the healthy members of this family or in any of the normal control subjects. The mutation leads to an amino acid change (G64W). This substitution is located at a corner of the modeled structure of CRYBA4, as shown in the modeled structure (Figure 4 and Figure 5).

## Discussion

In this study, we identified a mutation (c.225G>T) in exon 4 of *CRYBA4*. This mutation segregates within the proband and his father (the two patients of this family) and was not detected in normal members of this family or in 100 healthy controls. We conclude that this sequence change results in the onset of congenital cataract and microcornea in this family.

Three major classes of crystallins are found in the mammalian lens [28]. They are α-crystallin (40% of total crystalline protein), β-crystallin (35%), and γ-crystallin (25%) [9]. The β- and γ-crystallins are members of a superfamily as they share a common two-domain structure composed of four “Greek key” motifs, two in the NH_2_- and two in the COOH-terminal domain [29].The β-crystallins are major constituents of the human lens and include three basic and four acidic protein forms. Each subgroup is encoded by three genes (*CRYBA1*, *CRYBA2*, and *CRYBA4*; *CRYBB1*, *CRYBB2*, and *CRYBB3*) [30].The protein encoded by *CRYBA4* (belonging to the *CRBA* genes) contains 196 amino acids, and constitutes approximately 5% of the total soluble proteins in the young human lens [31].

β-Crystallins are expressed not only at the early developmental stages of the eye lens but also after birth. The temporal expression of crystallin genes vary in development. Different CRYB proteins can be found in both prenatal and postnatal developing lens; furthermore, they interact with each other [32,33]. Previous studies found homozygous changes in CRYBB2 that were associated with severe microphthalmia and cataract and found an interaction of CRYBB2–CRYBA4 monomers [34,35]. Human βA4-crystallin readily oligomerizes with human βB1-crystallin, a hetero-oligomer that can be purified [36].

**Figure 6:**
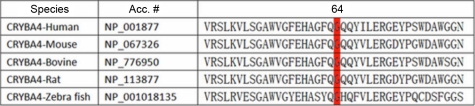
Interspecies sequence alignment of a portion of the CRYBA4 (crystallin, beta A4) amino acid sequence. The alignment data indicate that glycine at position 64 (indicated by the red background) is highly conserved in CRYBA4 of different species.

To date, Billingsley et al. [9] have reported two mutations (c.317T>C and c.242T>C) in exon 4 of *CRYBA4* by genetic analysis of a large Indian family with an autosomal dominant cataract phenotype. It is worth noting that one of the two mutations (c.242T>C) and the mutation reported in this study (c.225G>T) are in a highly conserved area of *CRYB* exon 4 (Figure 6), which indicates that the sequence changes in this area play an important role in the onset of congenital cataract.

The mutation described in this study leads to replacement of glycine by tryptophan at codon 64. From the modeled structure of CRYBA4 (Figure 4), which was built using special software (Swiss-PdbViewer 4.0.1; Torsten Schwede et al., Basel, Switzerland), we can report that this substitution takes place at a corner of the backbone structure. Moreover, previous studies support that glycine is often found in β-sheet secondary structures and is the amino acid appearing most frequently at position i+2 of β-turn [37,38]. Based on the above, we surmise that glycine at codon 64 probably forms similar secondary structures as β-sheet or β-turn. The substitution may result in damage to forming normal secondary structure during the CRYBA4 protein folding process so that the structure of the protein has reduced stability in the patient’s lens (Figure 4 and Figure 5). These series of changes may lead to disturbance of the lens transparency and functional integrity, resulting in cataract.

In the present study, we reported a novel missense mutation in two patients with congenital cataract and microcornea that come from the same Chinese family. This is the first report linking mutations in *CRYBA4* to cataractogenesis and microcornea. Our findings expand the mutation spectrum of *CRYBA4* and provide useful information in the study of molecular pathogenesis of congenital cataract.
